# Comparative study of endoscopy vs. transjugular intrahepatic portosystemic shunt in the management of gastric variceal bleeding

**DOI:** 10.1093/gastro/gou095

**Published:** 2015-02-02

**Authors:** Gursimran Singh Kochhar, Udayakumar Navaneethan, Jason Hartman, Jose Mari Parungao, Rocio Lopez, Ranjan Gupta, Baljendra Kapoor, Paresh Mehta, Madhu Sanaka

**Affiliations:** ^1^Department of Gastroenterology and Hepatology, Cleveland Clinic Foundation, Cleveland, OH, USA, ^2^Case Western School of Medicine, Cleveland, OH, USA and ^3^Department of Vascular and Interventional Radiology, Cleveland Clinic Foundation, Cleveland, OH, USA

**Keywords:** transjugular intrahepatic portosystemic shunt (TIPS), cyanoacrylate injection, gastric varices, gastrointestinal bleeding

## Abstract

**Background and Aim:** Gastric varices are associated with high mortality. There have been conflicting reports on whether endoscopic treatment with cyanoacrylate or the placement of a transjugular intrahepatic portosystemic shunt (TIPS) is more effective in the treatment of gastric varices. We compared the outcomes of patients treated with cyanoacrylate glue or TIPS for the management of acute gastric variceal bleeding.

**Methods:** The study was designed as a retrospective cohort analysis of patients undergoing either TIPS or endoscopic treatment with cyanoacrylate for acute gastric variceal bleeding at our institution from 2001 to 2011. Primary compared to studied between the two treatment modalities were the short-term treatment outcomes, including re-bleeding within 30 days, length of hospital stay and in-hospital mortality. Kaplan-Meier survival analysis was performed to assess factors associated with in-hospital mortality.

**Results:** A total of 169 patients were included in the analysis. The TIPS arm contained 140 patients and the cyanoacrylate arm contained 29 patients. There was no evidence to suggest any significant differences in demographics or disease severity. There were no differences between the TIPS arm and the cyanoacrylate armtwo groups in treatment outcomes including re-bleeding within 30 days (17.4% *vs.* 17.2%; *P* = 0.98), median length of stay in the hospital (4.5 days *vs.* 6.0 days; *P* = 0.35) or in-hospital mortality (9.0% *vs.* 11.1%; *P* = 0.74). In-hospital mortality was evaluated for 149 patients and lower albumin (*P* = 0.015), higher MELD score (*P < *0.001), higher CTP score (*P* = 0.005) and bleeding (*P* = 0.008) were all significantly associated with in-hospital death.

**Conclusion:** These findings suggest that both treatments are equally effective. Cyanoacrylate offers a safe, effective alternative to TIPS for gastric varices, and physician may choose the best therapy for each patient, factoring in the availability of TIPS or cyanoacrylate, the individual patient’s presentation, and cost.

## Introduction

Gastric varices may occur in the cardia or fundus of the stomach in 5–33% of patients with portal hypertension [1, 2]. Gastric varices are classified into several types: (i) gastric-esophageal varices (GOV)-1 and GOV-2, (ii) concurrent with esophageal varices and isolated gastric varices (IGV)-1 and IGV-2 and (iii) independent from esophageal varices [2]. Gastric variceal bleeds are rare, but tend to be severe when they occur and are thus associated with high mortality [3–5].

Gastric varices are managed by the use of cyanoacrylate injections or transjugular intrahepatic portosystemic shunt (TIPS). Although cyanoacrylate injection is not approved for use in the United States, it is widely used in other countries and a number of studies have demonstrated it to be safe and effective [6–8]. Some have recommended it be the first-line therapy for gastric variceal bleeding [9]. It has also been shown to be more effective than alcohol injection or band ligation [10, 11]. An international consensus meeting in 2005 found glue to be the only agent that should be recommended for controlling fundic gastric varices [12].

Another frequently employed treatment option is the insertion of a TIPS, which has been proven to be a safe and effective means of relieving portal hypertension, with a success rate as high as 100% [13, 14]. Uncontrolled trials have suggested that TIPS is an effective treatment for gastric varices in patients who failed to respond to initial endoscopic therapy [15, 16]. While both TIPS and cyanoacrylate have been proven effective in the treatment of gastric varices, there have been a number of conflicting reports on which is more effective [17–20].

The current American Association for the Study of Liver Disease (AASLD) recommendation for treating bleeding gastric fundal varices is to use endoscopic variceal obturation with tissue adhesives, such as cyanoacrylate, as first line and consider TIPS if cyanoacrylate is unavailable or if the bleeding cannot be controlled or recurs despite combined pharmacological and endoscopic therapy [1]. However, the AASLD cautions that these recommendations are based on relatively few studies.

The aim of our study was therefore to compare short-term treatment outcomes, including re-bleeding and survival, in patients with bleeding gastric varices treated with TIPS or endoscopic N-butyl-2-cyanoacrylate.

## Patients and Methods

### Patient selection

The study was designed as a retrospective cohort analysis. Approval was granted by the Cleveland Clinic Institutional Review Board (IRB). Data was gathered using electronic medical records between 2001 and 2011 that were part of an established IRB-approved database of all patients having undergone TIPS at our institution. A separate database of electronic medical records between 2001 and 2011, of all patients receiving treatment with cyanoacrylate at our institution was also utilized. Data was collected on patient demographics, clinical findings, procedural information, and treatment outcomes.

Patients were included if they had gastric varices as the source of bleeding on upper endoscopy and underwent treatment with either glue or TIPS. This was determined using International Classification of Diseases 9^th^ Revision (ICD-9) procedure codes. Patients were excluded if they had gastric varices but did not receive any therapy. Patients were excluded from the portion of the analysis assessing in-hospital death if they died at an unknown time. Patients meeting the inclusion criteria were divided into two groups: group A, which underwent treatment with TIPS, and group B, which underwent treatment with cyanoacrylate.

### Procedures

All TIPS were performed by experienced, fellowship-trained interventional radiologists. TIPS performed at our institution after 2004 made use of covered stents. Injection of cyanoacrylate was performed by advanced therapeutic endoscopists. In all cases, N-butyl-2-cyanoacrylate glue (Histoacryl, B Braun, Germany) was used. All gastric varices treated at our center with cyanoacrylate were GOV-2 based on Sarin’s classification system [2]. It is routine protocol to inject 2 mL of glue into the gastric varices. In patients who had endoscopic control of bleeding, endoscopic sessions were repeated with in 2-3 weeks for repeat treatment until complete obliteration of gastric varices was achieved.

### Data collection

Data was collected on demographic and clinical variables including age, gender, race, alcohol use, tobacco use, etiology of cirrhosis/portal hypertension, infection with hepatitis B or hepatitis C, use of proton pump inhibitors (PPIs), use of octreotide, use of antibiotics, and etiology of gastric varices. We also noted the use of other endoscopic therapies, whether or not hemostasis (immediate cessation of bleeding) was achieved, presence of concurrent esophageal varices, arteriovenous malformations, bleeding, spontaneous bacterial peritonitis (SBP), hepatic venous pressure gradient (HVPG), fresh frozen plasma, need for platelet transfusions, the need for blood transfusions, and the indication for TIPS.

Laboratory data was collected including albumin, bilirubin, blood urea nitrogen, serum creatinine, hemoglobin, platelets, international normalized ratio (INR), partial thromboplastin time (PTT), presence and severity of ascites, presence and grade of encephalopathy, model for end-stage liver disease (MELD) score, Child-Turcotte-Pugh (CTP) score and Child-Pugh class (A, B, or C).

### Clinical outcomes

Our primary outcomes of interest were short-term treatment outcomes, including re-bleeding within 30 days, re-bleeding in the hospital, need for airway intubation, length of stay in the hospital, acute kidney injury (AKI) following the procedure, in-hospital mortality, and days from procedure until death. We also examined whether or not patients had a repeat esophago-gastroduodenoscopy (EGD) and the indication for the same. Our secondary outcome of interest was to assess in-hospital mortality, particularly disease factors associated with in-hospital death.

### Statistical analysis

A univariate analysis was performed to compare the two treatment modalities; analysis of variance (ANOVA) or the non-parametric Kruskal-Wallis tests were used for continuous or ordinal variables and Pearson’s chi-squared test or Fisher’s exact test were used for categorical factors.

In addition, survival analysis was performed to assess in-hospital mortality. Follow-up time was defined as number of days from procedure to either death or discharge. Kaplan-Meier plots with log-rank tests were constructed and univariate Cox regression analysis was performed to assess factors associated with in-hospital mortality. Since only 14 deaths were observed in hospital, no multivariable analysis was done.

A value of *P < *0.05 was considered statistically significant. SAS (version 9.2, The SAS Institute, Cary, NC, USA) was used to perform all analyses. Data are presented as mean ± standard deviation, median [P25, P75] or No. (%).

## Results

### Characteristics of the study population

**Figure 1.**
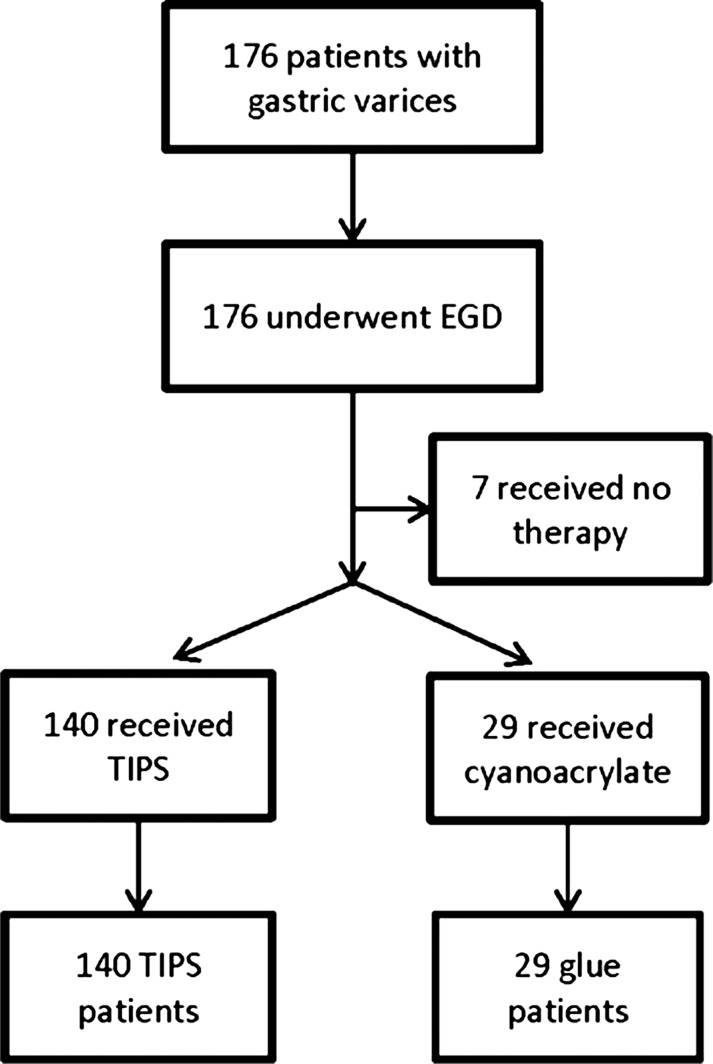
Algorithm of case selection

A total of 169 patients was included in the analysis. The TIPS arm contained 140 patients while the cyanoacrylate arm contained 29 (Figure 1). Seven patients were excluded as they did not undergo any therapy; in these cases the gastric varices visualized were judged too small to treat.

Patient demographics are summarized in Table 1. Average age was 56 ± 12 years and 62% were male. There were no statistically significant differences between the two groups. Disease factors are presented in Table 2. The most common liver disease etiology was hepatitis (31.0%) followed by alcoholic liver disease (21.9%), cryptogenic disease (14.8%), and non-alcoholic steatohepatitis (NASH) (13.5%). There were no significant differences between the cyanoacrylate arm and the TIPS arm in MELD scores (14.5 ± 9.2 *vs*.** 13.4 ± 6.4; *P* = 0.43), CTP scores (8.0 ± 2.6 *vs*.** 7.8 ± 2.0; *P* = 0.68), or Child-Pugh classes (*P* = 0.74). The only disease factor significantly different between the two groups was disease etiology, with subjects who underwent TIPS more likely to have alcoholic liver disease (24.6% *vs*.** 8.0%) or cryptogenic disease (16.9% *vs*.** 4.0%; *P < *0.001).

**Table 1.** Patients' demographics and personal history

Factor	Total (*n* = 169)	EGD only (*n* = 29)		EGD + TIPS (*n* = 140)		*P*-value
		*n*	Summary	*n*	Summary	
Age (years)	56.3 ± 12.0	29	56.9 ± 12.0	140	56.2 ± 12.0	0.75^a^
Male	105 (62.1)	29	15 (51.7)	140	90 (64.3)	0.20^b^
Race		29		140		0.35^b^
Caucasian	142 (84.0)		23 (79.3)		119 (85.0)	
African-American	16 (9.5)		3 (10.3)		13 (9.3)	
Hispanic	3 (1.8)		0 (0.0)		3 (2.1)	
Other	8 (4.7)		3 (10.3)		5 (3.6)	
Alcohol	68 (47.6)	28	15 (53.6)	115	53 (46.1)	0.48^b^
Smoking	96 (65.8)	28	21 (75.0)	118	75 (63.6)	0.25^b^
HIV	1 (1.1)	9	1 (11.1)	79	0 (0.0)	0.10^c^
Hepatitis B virus	20 (15.4)	18	5 (27.8)	112	15 (13.4)	0.12^b^
Hepatitis C virus	45 (33.3)	17	6 (35.3)	118	39 (33.1)	0.85^b^
Prior PPIs	74 (53.6)	29	18 (62.1)	109	56 (51.4)	0.30^b^
PPIs during EGD	87 (61.7)	29	21 (72.4)	112	66 (58.9)	0.18^b^
Octreotide	81 (55.1)	29	17 (58.6)	118	64 (54.2)	0.67^b^
Antibiotics	76 (51.7)	29	18 (62.1)	118	58 (49.2)	0.21^b^

Values presented as mean ± SD or *n* (%).

^a^ANOVA

^b^Pearson’s chi-squared test

^c^Fisher’s exact test.

EGD = esophago-gastroduodenoscopy; HIV = human immunodeficiency virus; PPIs = proton pump inhibitors; TIPS = transjugular intrahepatic portosystemic shunt

**Table 2.** Disease factors

Factor	Total (*n* = 169)	EGD only (*n* = 29)		EGD + TIPS (*n* = 140)		*P*-value
		*n*	Summary	*n*	Summary	
Etiology		25		130		** <0.001****^c^**
Hepatitis	48 (31.0)		7 (28.0)		41 (31.5)	
Alcoholic liver disease	34 (21.9)		2 (8.0)		32 (24.6)	
Cryptogenic disease	23 (14.8)		1 (4.0)		22 (16.9)	
Non-alcoholic steatohepatitis	21 (13.5)		5 (20.0)		16 (12.3)	
Primary sclerosing cholangitis	8 (5.2)		0 (0.0)		8 (6.2)	
Primary biliary cirrhosis	6 (3.9)		1 (4.0)		5 (3.8)	
Others	15 (9.7)		9 (36.0)		6 (4.6)	
Ascites		29		133		0.26^b^
None	89 (54.9)		19 (65.5)		70 (52.6)	
Mild	25 (15.4)		3 (10.3)		22 (16.5)	
Moderate/Severe	48 (29.6)		7 (24.1)		41 (30.8)	
Encephalopathy		29		132		0.53^b^
None	134 (83.2)		23 (79.3)		111 (84.1)	
Grade 1–2	23 (14.3)		5 (17.2)		18 (13.6)	
Grade 3–4	4 (2.5)		1 (3.4)		3 (2.3)	
MELD score	13.6 ± 7.0	28	14.5 ± 9.2	128	13.4 ± 6.4	0.43^a^
CTP score	7.8 ± 2.1	28	8.0 ± 2.6	125	7.8 ± 2.0	0.68^a^
Child-Pugh class		28		125		0.74^b^
A	48 (31.4)		11 (39.3)		37 (29.6)	
B	74 (48.4)		10 (35.7)		64 (51.2)	
C	31 (20.3)		7 (25.0)		24 (19.2)	

Values presented as mean ± SD or *n* (%).

^a^Kruskal-Wallis test

^b^Pearson’s chi-squared test

^c^Fisher’s exact test.

CTP = Child-Turcotte-Pugh; EGD = esophago-gastroduodenoscopy; MELD = model for end-stage liver disease; TIPS = transjugular intrahepatic portosystemic shunt

### Treatment details

Table 3 presents a summary of EGD outcomes and details by treatment group. Twenty-nine of the 140 patients who underwent TIPS also underwent treatment with various EGD therapies; seven patients received sclerotherapy, four received epinephrine, two received glue, eight received banding, four received clips, and four underwent balloon-occluded retrograde transvenous obliteration. Hemostatis was achieved in 100% of those who received glue, as opposed to 59.1% of those who underwent TIPS (*P* = 0.031)

**Table 3.** EGD findings

Factor	Total (*n* = 169)	EGD only (*n* = 29)		EGD + TIPS (*n* = 140)		*P*-value
		*n*	Summary	*n*	Summary	
Therapy		29		139		
Sclerotherapy	7 (4.2)		0 (0.0)		7 (5.0)	0.22^c^
Epinephrine	4 (2.4)		0 (0.0)		4 (2.9)	0.99^c^
Glue	31 (18.5)		29 (100.0)		2 (1.4)	**<0.001****^b^**
Banding	8 (4.8)		0 (0.0)		8 (5.8)	0.19^c^
Clips	4 (2.4)		0 (0.0)		4 (2.9)	0.99^c^
Balloon	4 (2.4)		0 (0.0)		4 (2.9)	0.99^c^
Number of EGD therapies		29		139		**<0.001****^a^**
0	116 (69.0)		0 (0.0)		116 (83.5)	
1	46 (27.4)		29 (100.0)		17 (12.2)	
2	6 (3.6)		0 (0.0)		6 (4.3)	
Hemostasis	21 (70.0)	8	8 (100.0)	22	13 (59.1)	**0.031****^c^**
Esophageal varices	117 (69.2)	29	17 (58.6)	140	100 (71.4)	0.17b
Arteriovenous malformations	1 (0.59)	29	0 (0.0)	140	1 (0.71)	0.99c
Bleeding	26 (15.5)	29	5 (17.2)	139	21 (15.1)	0.77b
SBP	5 (3.1)	29	2 (6.9)	134	3 (2.2)	0.22c
HVPG	16 [11, 21]	1	12 [12, 12]	95	16 [11, 21]	0.48a
Fresh frozen plasma	28 (19.0)	28	6 (21.4)	119	22 (18.5)	0.72b
Platelets transfusion	17 (11.6)	28	2 (7.1)	119	15 (12.6)	0.42^b^
Blood transfusion	81 (53.6)	28	14 (50.0)	123	67 (54.5)	0.67^b^

Values presented as median [P25, P75] or *n* (%).

^a^Kruskal-Wallis test

^b^Pearson's chi-squared test

^c^Fisher's exact test.

EGD = esophago-gastroduodenoscopy; HVPG = hepatic venous pressure gradient;

SBP = spontaneous bacterial peritonitis; TIPS = transjugular intrahepatic portosystemic shunt.

### Treatment outcomes

For treatment outcomes, the primary endpoint of this study, there were no significant differences between the two groups (Table 4). Re-bleeding rates were 10.3% within the hospital stay and 17.2% within 30 days in the cyanoacrylate arm and 13.8% and 17.4% in the TIPS arm (*P* = 0.62 and *P* = 0.98, respectively). Median length of stay was 6.0 days in the cyanoacrylate arm and 4.5 days in the TIPS arm (*P* = 0.35). We detected no significant differences in immediate post-procedural complications, including the need for intubation (*P* = 0.18), AKI (*P* = 0.14), or encephalopathy (*P* = 0.53). A large number of patients in both groups (55.0%) underwent repeat EGDs for follow-up (60.2%), re-bleeding (32.3%), and surveillance (7.5%); there were no significant differences in receiving repeat EGD (*P* = 0.21) or its indication (*P* = 0.17).

**Table 4.** Treatment outcomes

Factor	Total (*n* = 169)	EGD only (*n* = 29)		EGD + TIPS (*n* = 140)		*P*-value
		*n*	Summary	*n*	Summary	
Re-bleed in 30 days	29 (17.4)	29	5 (17.2)	138	24 (17.4)	0.98^b^
Re-bleed in hospital	22 (13.2)	29	3 (10.3)	138	19 (13.8)	0.62^b^
Intubation	39 (24.7)	29	10 (34.5)	129	29 (22.5)	0.18^b^
Encephalopathy		29		132		0.53^a^
None	134 (83.2)		23 (79.3)		111 (84.1)	
Grade 1–2	23 (14.3)		5 (17.2)		18 (13.6)	
Grade 3–4	4 (2.5)		1 (3.4)		3 (2.3)	
Acute kidney injury	25 (15.2)	29	7 (24.1)	136	18 (13.2)	0.14^b^
Repeat EGD	93 (55.0)	29	19 (65.5)	140	74 (52.9)	0.21^b^
Indication		19		74		0.17^b^
Follow-up EGD	56 (60.2)		15 (78.9)		41 (55.4)	
Re-bleed/Hemetemesis	30 (32.3)		3 (15.8)		27 (36.5)	
Surveillance	7 (7.5)		1 (5.3)		6 (8.1)	
Length of stay (days)	5 [0, 10]	29	6 [3, 9]	138	4.5 [0, 10]	0.35^a^
In-hospital mortality	14 (9.4)	27	3 (11.1)	122	11 (9.0)	0.74^b^

Values presented as Median [P25, P75] or *n* (%).

^a^Kruskal-Wallis test

^b^Pearson’s chi-squared test

^c^Fisher’s exact test.

EGD = esophago-gastroduodenoscopy; TIPS = transjugular intrahepatic portosystemic shunt

Our secondary endpoints of mortality assessment included 149 patients: 20 who died at an unknown time were excluded from this part of the analysis. Fourteen patients died while in the hospital prior to being discharged and 135 were discharged home at 30 days. Table 5 presents disease factors associated with in-hospital mortality. Table 6 compares EGD findings and short-term outcomes between those who were discharged alive and those who died within the hospital. Significant associations were seen with increased bleeding (HR 4.2; *P* = 0.008) and increased HVPG (HR 1.10; *P* = 0.033) in those who died in the hospital. Those who died in the hospital were more likely to undergo intubation (HR 6.8; *P* = 0.014) and experience AKI (HR 8.2; *P* = 0.002).

**Table 5.** In-hospital death and disease factors

Factor	Discharged alive (*n* = 135)		In-hospital death (*n* = 14)		Hazard Ratio (95% CI)	*P*-value
	n	Summary	n	Summary		
Etiology	122		14			
Hepatitis		35 (28.7)		7 (50.0)	Reference	
Alcoholic liver disease		26 (21.3)		3 (21.4)	0.34 (0.08, 1.4)	0.13
Cryptogenic disease		18 (14.8)		2 (14.3)	0.46 (0.09, 2.3)	0.33
NASH		17 (13.9)		1 (7.1)	0.16 (0.02, 1.3)	0.09
Others		8 (6.6)		0 (0.0)	0.16 (0.02, 1.3)	0.091
Albumin	129	3.1 ± 0.7	14	2.2 ± 0.8	0.34 (0.14,0.81)	**0.015**
Bilirubin	129	1.4 [0.9, 2.6]	14	11.0 [4.2, 17.3]	1.07 (1.02, 1.1)	**0.007**
Blood urea nitrogen	131	16 [11, 26]	14	34 [27, 60]	1.02 (1.01, 1.03)	**<0.001**
Serum creatinine	131	0.86 [0.70, 1.2]	14	1.5 [1.2, 2.9]	1.6 (1.2, 2.3)	**0.004**
Hemoglobin	134	10.3 ± 2.3	14	8.5 ± 2.1	0.75 (0.55, 1.02)	0.064
Platelets	130	84.5 [57, 142]	14	64.5 [37, 92]	0.99 (0.98, 1.00)	0.13
INR	132	1.2 ± 0.24	14	2.0 ± 0.84	3.7 (2.1, 6.5)	**<0.001**
Partial thromboplastin time	121	30.9 [27.5, 34.7]	14	43.3 [34.4, 63.6]	1.2 (1.04, 1.3)^a^	**0.009**
Ascites	132		12		0.93 (0.49, 1.8)	0.83
None		74 (56.1)		5 (41.7)		
Mild		20 (15.2)		1 (8.3)		
Moderate/severe		38 (28.8)		6 (50.0)		
Encephalopathy	131		11		2.1 (0.99,4.6)	0.052
None		114 (87.0)		4 (36.4)		
Grade 1–2		14 (10.7)		6 (54.5)		
Grade 3–4		3 (2.3)		1 (9.1)		
MELD score	124	12.0 ± 5.1	14	27.0 ± 9.0	1.1 (1.08, 1.2)	**<0.001**
CTP score	124	7.5 ± 1.9	11	11.0 ± 1.8	1.5 (1.1, 2.0)	**0.005**
Child-Pugh class	124		11		4.0 (1.2, 13.4)	**0.025**
A		46 (37.1)		0 (0.0)		
B		59 (47.6)		3 (27.3)		
C		19 (15.3)		8 (72.7)		

Values presented as mean ± SD, median [P25, P75] or *n* (%).

Hazard ratios and *P*-values correspond to univariate Cox regression analysis.

CTP = Child-Turcotte-Pugh; INR = international normalized ratio; MELD = model for end-stage liver disease; NASH = non-alcoholic steatohepatitis;

^a^HR corresponds to a 10-unit increase in partial thromboplastin time.

**Table 6.** In-hospital death and EGD findings and treatment outcomes

Factor	Discharged alive (*n* = 135)		In-hospital death (*n* = 14)		Hazard Ratio (95% CI)	*P*-value
	*n*	Summary	*n*	Summary		
Treatment	135		14		0.96 (0.27, 3.4)	0.95
EGD only		24 (17.8)		3 (21.4)		
EGD + TIPS		111 (82.2)		11 (78.6)		
Sclerotherapy	134	6 (4.5)	14	1 (7.1)	0.78 (0.10, 6.0)	0.82
Glue	134	25 (18.7)	14	4 (28.6)	1.3 (0.40, 4.1)	0.67
Banding	134	7 (5.2)	14	1 (7.1)	2.1 (0.26, 16.3)	0.49
Number of EGD therapies	134		14		1.3 (0.62, 2.9)	0.45
0		92 (68.7)		6 (42.9)		
1		37 (27.6)		7 (50.0)		
2		5 (3.7)		1 (7.1)		
Hemostasis	21	17 (81.0)	8	4 (50.0)	0.34 (0.08, 1.4)	0.13
Esophageal varices	135	94 (69.6)	14	9 (64.3)	0.79 (0.26, 2.4)	0.67
Bleeding	134	17 (12.7)	14	8 (57.1)	4.2 (1.5, 12.3)	**0.008**
SBP	131	2 (1.5)	13	3 (23.1)	3.6 (0.95, 13.7)	0.059
HVPG	74	15 [11, 21]	8	19.5 [14, 23.5]	1.10 (1.01, 1.2)	**0.033**
Fresh frozen plasma	122	21 (17.2)	8	5 (62.5)	2.5 (0.56, 11.3)	0.23
Platelets transfusion	122	12 (9.8)	8	4 (50.0)	3.3 (0.80, 13.6)	0.099
Blood transfusion	121	61 (50.4)	11	8 (72.7)	0.68 (0.17, 2.7)	0.59
Re-bleed in 30 days	133	22 (16.5)	14	5 (35.7)	1.3 (0.44, 4.1)	0.6
Re-bleed in hospital	133	16 (12.0)	14	5 (35.7)	1.5 (0.50, 4.7)	0.45
Intubation	126	26 (20.6)	12	10 (83.3)	6.8 (1.5, 31.7)	**0.014**
Acute kidney injury	132	13 (9.8)	13	10 (76.9)	8.2 (2.2, 31.1)	**0.002**
Length of stay (days)	134	4 [0, 9]	14	7.5 [4, 18]	0.87 (0.75, 1.00)	0.055

Values presented as median [P25, P75] or *n* (%)

Hazard ratios and *P*-values correspond to univariate Cox regression analysis.

EGD = esophago-gastroduodenoscopy; HVPG = hepatic venous pressure gradient; SBP = spontaneous bacterial peritonitis; TIPS = transjugular intrahepatic portosystemic shunt

## Discussion

Gastric varices, though rare, are associated with high mortality [3–5]. It has been suggested that the incidence may be increasing, possibly as a secondary effect of banding esophageal varices [21]; therefore determining the most effective means of treatment—insertion of a TIPS or injection with cyanoacrylates—has received considerable attention.

Our study, with 169 patients, is the largest to date on the comparative effectiveness of glue injection *vs*.** TIPS. The disease burden was not significantly different between the two groups; MELD scores in particular were equivalent. We found no differences in re-bleeding rates, survival, lengths of hospital stay, or post-procedural complications. As there were no significant differences in demographics or disease severity between the two groups, our findings suggest that there is no difference in treatment outcomes between TIPS and treatment with cyanoacrylate.

To date, the only randomized, controlled trial comparing TIPS and cyanoacrylate concluded TIPS to be more effective, based on a lower rate of re-bleeding from gastric varices (11% *vs*.** 38%; *P* = 0.014) [17]. Overall survival and rates of complications were similar in both groups. Retrospective cohort studies have found that those who underwent treatment with glue had a shorter hospital course (13 days *vs*.** 18 days; *P* = 0.05) [19], and that patients treated with cyanoacrylate had similar re-bleeding and survival rates, as well as less procedure-related morbidity [18, 20]. In addition, 41% of patients with a TIPS required re-hospitalization, against 1.6% of patients who were treated with glue (*P < *0.0001) [18]. All previous studies agree that the choice of glue or TIPS does not seem to influence overall survival.

Our study agrees with the results of previous retrospective studies—that treatment with TIPS or cyanoacrylate results in equivalent rates of acute complication and survival. We did not detect the shorter hospital stay in the cyanoacrylate arm, found by previous studies [22]. The median length of stay in both groups was also much shorter in our study (5 days for both groups). We did not observe any significant difference in re-bleeding rates between the two groups, whereas Lo *et al*.** [17] found that those treated with glue were more likely to have re-bleeding. One possible explanation for the difference in outcomes is that, in their study, 17 out of 37 patients in the cyanoacrylate arm had GOV1-type varices, whereas all of our patients treated with cyanoacrylate had GOV2-type. In addition, liver disease etiology differed between their population and ours. In their population, disease etiology was 78% hepatitis, 16% alcoholic, and 6% cryptogenic whereas, in ours, etiology was 31.0% hepatitis, 21.9% alcoholic, 14.8% cryptogenic, and 13.5% NASH. This suggests that our results may be more applicable to a North American population. Our study agrees with earlier studies, that the choice of glue or TIPS does not seem to affect overall survival; we found in-hospital mortality and overall mortality to be high in both of our study groups.

This study is limited by its retrospective nature and the use of databases, which may have introduced confounds such as incorrect coding. Furthermore this is a single-institutional study, which limits the general applicability of our findings. In addition, not all of the relevant data we wished to collect was recorded in the medical records. It has also been suggested that the type of gastric varices is also an important consideration, as GOV1 may be considered closer to esophageal varices and therefore easier to control with sclerotherapy [4]. Information on the incidence of bacteremia was also not available. In addition, it has been suggested that TIPS should always be paired with embolization of gastric varices [14, 23], however, this was not always done in our study population. The results of our study suggest that it may be useful to carry out a randomized, controlled trial across multiple North American institutions, comparing treatment of GOV2-type varices with covered TIPS paired with embolization against endoscopic cyanoacrylate therapy.

### Conclusion

Whether TIPS or EGD is superior in managing gastric varices remains a subject of much debate. However, our results suggest that patients with equivalent disease did equally well with either treatment. Therefore, with two equally effective treatment options, the ordering provider may decide which treatment is most appropriate based on several factors, including the availability of TIPS insertion by experienced interventional radiologists or the availability of cyanoacrylate by advanced endoscopists. For patients with hepatic encephalopathy where TIPS may be contraindicated, cyanoacrylate offers an alternative treatment. Finally, with current increased focus on efficient health-care spending, the higher cost of TIPS should be considered in the light of similar outcomes from both treatments.

*Conflict of interest statement.* none declared.
